# Association of the triglyceride-glucose index with early-onset atherosclerotic cardiovascular disease events and all-cause mortality: a prospective cohort study

**DOI:** 10.1186/s12933-024-02249-4

**Published:** 2024-04-29

**Authors:** Qiqi Hou, Qi Qi, Quanle Han, Jie Yu, Jianmei Wu, Hui Yang, Shuohua Chen, Shouling Wu, Kangbo Li

**Affiliations:** 1 Department of Cardiology, Tangshan Gongren Hospital, Tangshan, China; 2 Hebei Medical University, Shijiazhuang, China; 3 Department of Cardiovascular Surgery, Tangshan Gongren Hospital, Tangshan, China; 4 Department of Psychology, Tangshan Gongren Hospital, Tangshan, China; 5 Department of Cardiology, Kailuan General Hospital, Tangshan, China; 6 School of Clinical Medicine, North China University of Science and Technology, Tangshan, China

**Keywords:** Triglyceride glucose index, Atherosclerotic cardiovascular disease events, All-cause mortality, Young and middle-aged people, North China

## Abstract

**Background:**

The association between the triglyceride glucose (TyG) index and the risk of early-onset atherosclerotic cardiovascular disease (ASCVD) events or all-cause mortality in young and middle-aged people is not fully elucidated.

**Methods:**

The present study included 64,489 young and middle-aged people who participated in the 2006 Kailuan Study physical examination. Multivariate Cox proportional hazards models and restricted cubic spline curves were used to assess the association of TyG index with early-onset ASCVD events and all-cause mortality.

**Results:**

During a median of 11-year follow-up, 1984 (3.08%) participants experienced at least one ASCVD event and 1,392 (2.16%) participants experienced all-cause death. A higher TyG index was significantly associated with a higher risk of early-onset ASCVD events (HR: 1.61, 95% CI 1.38–1.89) and all-cause mortality (HR: 1.39, 95% CI 1.17–1.65), respectively. For each unit increase in TyG index, the risk of early-onset ASCVD events increased by 20%. In addition, there was a non-linear association between the TyG index and early-onset ASCVD events (*P* for non-linear < 0.01), and a linear association between TyG index and all-cause mortality (*P* for non-linear = 0.476).

**Conclusions:**

A higher TyG index is significantly associated with an increased incidence of early-onset ASCVD events and all-cause mortality in a young and middle-aged population from North China.

## Introduction

Atherosclerotic cardiovascular disease (ASCVD) is a major contributor to the global burden of disease affecting over 523 million people globally [1, 2]. Current studies have identified insulin resistance (IR) is one of the most important risk factors for ASCVD [3]. It has been accepted that the hyperinsulinemic-euglycemic clamp technique is the gold standard for assessing IR, however it is not easy to apply this technique on a large scale in the clinic [4]. As a surrogate for IR, the triglyceride-glucose (TyG) index is less expensive and easier to perform. Previous studies have demonstrated that the TyG index is significantly associated with the new-onset ASCVD events in the elderly [5–9]. However, no study has yet to be conducted to investigate the association between the TyG index and the risk of ASCVD events in a young and middle-aged population. Therefore, our study aimed to fill this gap by investigating the association between the TyG index and the risk of early-onset ASCVD events or all-cause mortality in a young and middle-aged population from North China.

## Methods

### Study participants

**Fig. 1:**
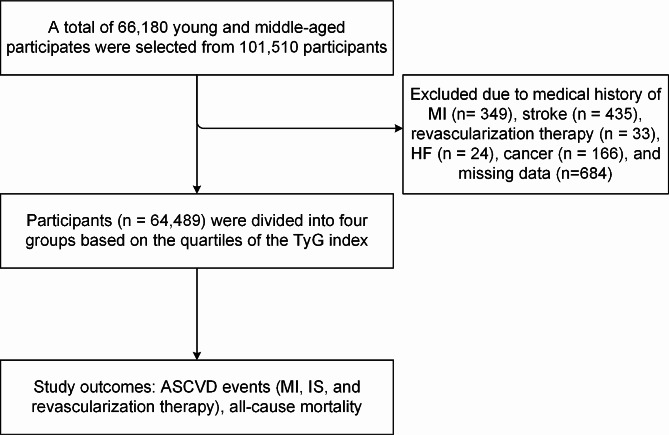
Flow chart for inclusion of study participants. *MI* myocardial infarction, *HF* heart failure, *IS* ischemic stroke, *TyG index* triglyceride-glucose index

The Kailuan Study is a prospective cohort study conducted in the community of Tangshan, China. The relevant design of the Kailuan study has been described in detail in previous articles [10–12]. Here, young and middle-aged participants were defined as males aged less than 55 years or females aged less than 65 years [13]. A total of 101,510 participants initially participated the Kailuan study in the year of 2006, among which, there are 66,180 young and middle-aged participates. We excluded participates who had experienced myocardial infarction (MI) (*n* = 349), stroke (*n* = 435), revascularization therapy (*n* = 33), heart failure (HF) (*n* = 24) and cancer (*n* = 166) before baseline enrollment. Except that, 684 subjects who lacked fasting blood glucose (FBG) or triglyceride (TG) data at baseline were further excluded. Finally, a total of 64,489 participants were enrolled in our current study (Fig. 1).

### Data collection and definitions

The participants’ demographic characteristics, lifestyle characteristics, medical history, and medication history were collected, respectively. The diagnosis of hypertension was based on a self-reported history of hypertension, a mean blood pressure ≥ 140/90 mmHg on two measurements at physical examination, or a normal blood pressure that was being treated with antihypertensive medication [14]. The diagnosis of diabetes mellitus was based on a self-reported history of diagnosed diabetes mellitus or a FBG ≥ 7.0 mmol/L or normal blood glucose but on hypoglycemic treatment [15]. Blood was collected after fasting for more than 8 h for every participant. Biochemical parameters consist of high-sensitivity C-reactive protein (hs-CRP), high-density lipoprotein cholesterol (HDL-C), total cholesterol (TC), TG, and low-density lipoprotein cholesterol (LDL-C) were analyzed using an automated analyzer (Hitachi, Tokyo, Japan). These parameters were monitored by professional quality control personnel to ensure accuracy.

### Calculation of the TyG index

The TyG index was calculated as ln [TG (mg/dL) × FBG (mg/dL)/2] [7]. Based on the quartiles of the TyG index, the subjects were divided into four groups: quartile 1 (Q1) (3.61 ≤ TyG < 8.17), quartile 2 (Q2) (8.17 ≤ TyG < 8.57), quartile 3 (Q3) (8.57 ≤ TyG < 9.06), and quartile 4 (Q4) (9.06 ≤ TyG ≤ 12.25).

### Outcomes and follow-up

The outcomes observed in this study were ASCVD events and all-cause mortality. Here, ASCVD events consist of MI, ischemic stroke (IS), and revascularization therapy [(coronary artery bypass grafting (CABG) and percutaneous coronary intervention (PCI)]. MI and IS were identified by ICD-10 codes, CABG and PCI were identified by the International Classification of Diseases, Ninth Revision, Clinical Modification (ICD-9-CM) codes. All-cause mortality refers to death from any cause. The state of life is obtained through inquiry by the Human Resources and Social Security Bureau of Hebei Province or through direct contact with the participant’s family members. All outcomes were reviewed by medical professionals.

Follow-up visits were conducted annually since the 2006 physical examination. Participants were followed until the first occurrence of any ASCVD events, death, or reaching 55 years old for males or 65 years old for females. The date of the last follow-up visit was December 31, 2021. When all-cause mortality was studied, it was considered as an independent outcome.

### Statistical analysis

All analyses were conducted using SAS version 9.4 (SAS Institute, Cary, NC, USA). Participants were divided into four groups based on the TyG index quartiles. Baseline characteristics are expressed as the mean ± standard deviation or as frequencies and percentages. Incidence densities per 1000 person-years were calculated based on follow-up time and number of events. Incidence curves for ASCVD events, subtypes, and all-cause mortality were plotted using the Kaplan–Meier method, and the log-rank test was used to assess between-group differences in incidence rates. Hazard ratios (HR) and 95% confidence intervals (95% CI) for composite ASCVD events, ASCVD events subtypes and all-cause mortality were calculated using Cox proportional hazards regression modeling, respectively. The proportional risk assumption was assessed by examining the Schoenfeld residuals. *P* values for trends were calculated using quartiles of changes in the TyG index as an ordinal variable. The robustness of the association between the TyG index and the risk of ASCVD events or all-cause mortality was also analyzed. First, the TyG index was included in the model as a continuous variable. Second, to rule out the potential effect of reverse causality, the main analyses were repeated, excluding participants who developed ASCVD events or all-cause mortality during the first 2 years of follow-up. Subgroup analyses were conducted after stratification by sex, BMI, presence of hypertension, presence of diabetes mellitus, smoking status, and drinking status. Likelihood ratios were used to test for the presence of multiplicative interactions between the TyG index and these variables. In addition, we examined the predictive value of the TyG index for early-onset ASCVD events and all-cause mortality by calculating the C-statistic, net reclassification index (NRI), and integrated discrimination improvement (IDI). A two-sided *P* < 0.05 was considered to statistical significance.

## Results

### Characteristics of the study population

The characteristics of 64,489 participants by quartile of the baseline TyG index are shown in Table 1. The patients enrolled in the study had a mean age of 44.9 ± 8.9 years and the percentage of male was 72.07%. The median (IQR) of the baseline TyG index was 8.57 (8.65 ± 0.70). Compared with the lowest quartile group, the higher TyG index groups have more elders, males, smokers and drinkers and with higher prevalence of hypertension, diabetes mellitus, higher usage rate of antihypertensive agents, antidiabetic agents, lipid-lowering agents, higher BMI, SBP, DBP, TC, LDL-C, hs-CRP, lower HDL-C (all *P* < 0.05).

**Table 1:** Baseline characteristics according to quartiles of the TyG index

Variables	Total (*n* = 64,489)	Quartile 1 (*n* = 16,121)	Quartile 2 (*n* = 16,122)	Quartile 3 (*n* = 16,124)	Quartile 4 (*n* = 16,122)	*P* value
Age, years	44.9 ± 8.9	43.2 ± 9.5	44.7 ± 8.9	45.6 ± 8.6	46.3 ± 8.1	< 0.0001
Male, n (%)	46,479 (72.07)	10,092 (62.60))	11,445 (70.99)	12,048 (74.72)	12,894 (79.98)	< 0.0001
BMI, kg/m^2^	25.04 ± 3.49	23.35 ± 3.18	24.63 ± 3.27	25.65 ± 3.32	26.52 ± 3.35	< 0.0001
SBP, mmHg	126.79 ± 19.34	119.83 ± 17.46	125.34 ± 18.40	128.70 ± 19.13	133.28 ± 19.76	< 0.0001
DBP, mmHg	82.70 ± 11.76	78.37 ± 10.71	81.95 ± 11.30	83.92 ± 11.50	86.55 ± 11.97	< 0.0001
TyG index	8.65 ± 0.70	7.83 ± 0.27	8.38 ± 0.11	8.80 ± 0.14	9.60 ± 0.47	< 0.0001
FBG, mmol/L	5.41 ± 1.55	4.83 ± 0.65	5.07 ± 0.72	5.39 ± 1.03	6.36 ± 2.51	< 0.0001
TC, mmol/L	4.93 ± 1.10	4.63 ± 0.87	4.89 ± 0.92	5.11 ± 0.98	5.10 ± 1.44	< 0.0001
TG, mmol/L	1.27 (0.89–1.96)	0.69 (0.57–0.80)	1.09 (0.98–1.21)	1.56 (1.37–1.79)	2.87 (2.27–4.05)	< 0.0001
HDL-C, mmol/L	1.53 ± 0.37	1.54 ± 0.38	1.54 ± 0.36	1.52 ± 0.36	1.52 ± 0.40	< 0.0001
LDL-C, mmol/L	2.36 ± 0.83	2.20 ± 0.85	2.39 ± 0.77	2.47 ± 0.79	2.37 ± 0.89	< 0.0001
hs-CRP, mg/L	0.70 (0.27–1.90)	0.50 (0.20–1.53)	0.64 (0.24–1.70)	0.77 (0.30–1.90)	0.93 (0.36–2.33)	< 0.0001
Hypertension, n (%)	23,384 (36.26)	3275 (20.32)	5296 (32.85)	6610 (40.99)	8203 (50.88)	< 0.0001
Diabetes mellitus, n (%)	4722 (7.32)	122 (0.76)	281 (1.74)	914 (5.67)	3405 (21.12)	< 0.0001
Smoking, n (%)	22,728 (35.24)	5213 (32.34)	5198 (32.24)	5771 (35.79)	6546 (40.60)	< 0.0001
Drinking, n (%)	25,038 (38.83)	6052 (37.54)	5715 (35.45)	6242 (38.71)	7029 (43.60)	< 0.0001
Snoring, n (%)	22,571 (35.00)	5348 (33.17)	5146 (31.92)	5723 (35.49)	6354 (39.41)	< 0.0001
High educational background, n (%)	15,318 (23.75)	4878 (30.26)	3625 (22.48)	3611 (22.40)	3204 (19.87)	< 0.0001
Antihypertensive agents, n (%)	4707 (7.30)	629 (3.90)	854 (5.30)	1384 (8.58)	1840 (11.41)	< 0.0001
Antidiabetic agents, n (%)	915 (1.42)	46 (0.29)	83 (0.51)	192 (1.19)	594 (3.68)	< 0.0001
Lipid-lowering agents, n (%)	381 (0.59)	35 (0.22)	66 (0.41)	90 (0.56)	190 (1.18)	< 0.0001

*BMI* body mass index, *SBP* systolic blood pressure, *DBP* diastolic blood pressure, *TyG index* triglyceride-glucose index, *FBG* fasting blood glucose, *TC* total cholesterol, *TG* triglyceride, *HDL-C* high density lipoproteincholesterol, *LDL-C* low density lipoproteincholesterol, *hs-CRP* high sensitivity C-reactive protein

### Association of the TyG index with ASCVD events and all-cause mortality

**Fig. 2:**
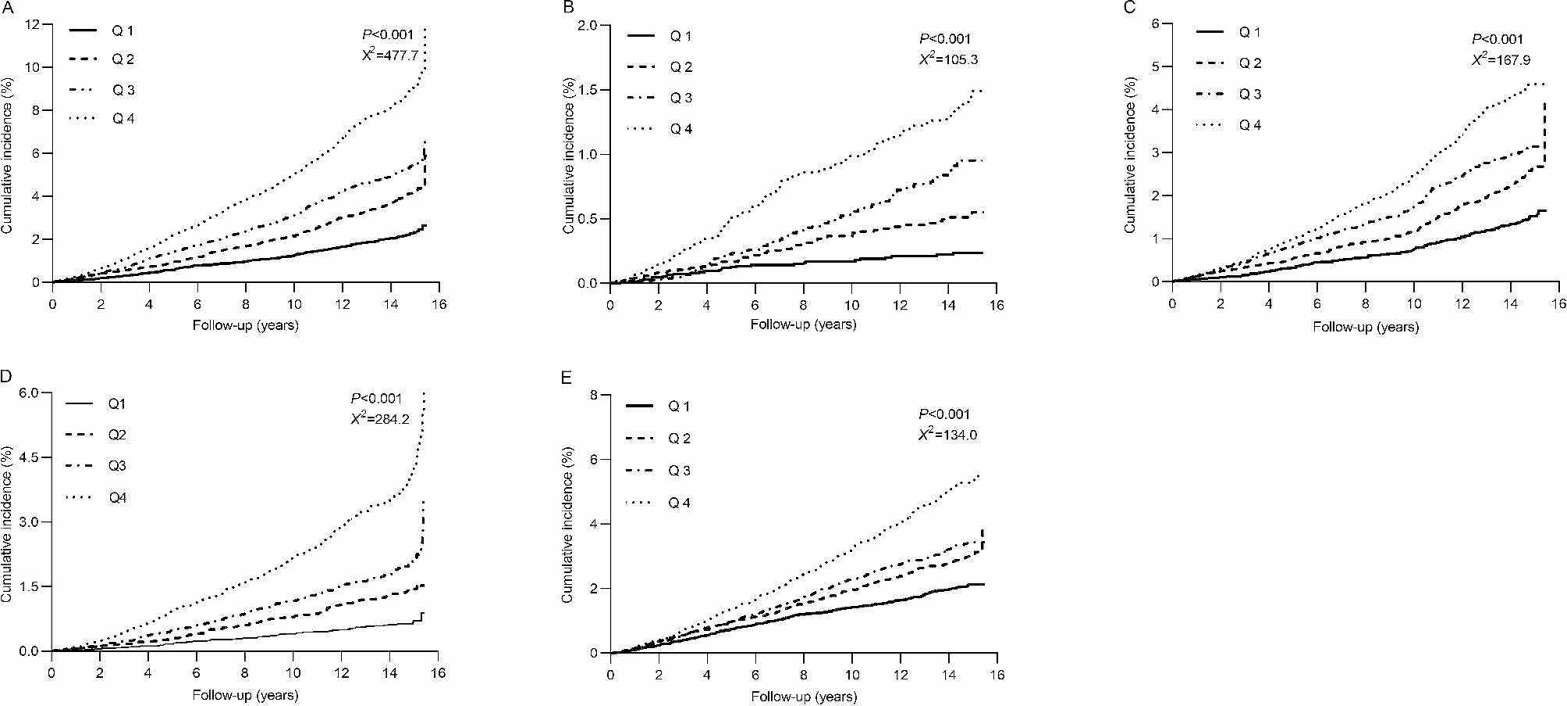
Cumulative incidence of **A** ASCVD events, **B** MI, **C** IS, **D** revascularization therapy, **E** all-cause mortality by quartiles of baseline TyG index. *ASCVD* atherosclerotic cardiovascular disease, *MI* myocardial infarction, *IS* ischemic stroke, *TyG index* triglyceride-glucose index

The median follow-up was 10.8 years (IQR 4.7–14.7 years). During the follow-up period, 1984 participants (3.08%) had composite ASCVD events, with 312 cases of MI, 1104 cases of IS, and 766 revascularization therapies, respectively. A total of 198 patients (0.31%) experienced two or more ASCVD events. In addition, a total of 1392 (2.16%) deaths occurred. The incidence of composite ASCVD events increased from 1.48 (1.31–1.67) per 1000 person-years in Q1 to 5.66 (5.28–6.06) per 1000 person-years in Q4. In addition, the incidence of all-cause mortality also increased from 1.42 (1.25–1.61) per 1000 person-years in Q1 to 3.38 (3.09–3.70) per 1000 person-years in Q4 (Table 2). In addition, the Kaplan-Meier curve revealed that the cumulative incidence of composite ASCVD events, ASCVD events subtypes, and all-cause mortality were higher in Q4 than in the other quartiles (Fig. 2).

**Table 2:** HR for risk of outcomes according to quartiles of the baseline TyG index

Variables	Quartile 1	Quartile 2	Quartile 3	Quartile 4	*P* for trend
ASCVD events	251	411	522	800	
Incidence, per 1000 person-year	1.48 (1.31–1.67)	2.62 (2.38–2.88)	3.49 (3.20–3.80)	5.66 (5.28–6.06)	
Model 1	Reference	1.53 (1.30–1.78)	1.85 (1.59–2.15)	2.77 (2.40–3.20)	< 0.001
Model 2	Reference	1.30 (1.11–1.52)	1.34 (1.15–1.57)	1.61 (1.38–1.89)	< 0.001
MI	29	57	87	139	
Incidence, per 1000 person-year	0.17 (0.12–0.24)	0.36 (0.28–0.47)	0.58 (0.47–0.71)	0.97 (0.82–1.14)	
Model 1	Reference	1.83 (1.17–2.86)	2.67 (1.75–4.07)	4.12 (2.75–6.16)	< 0.001
Model 2	Reference	1.57 (0.99–2.46)	1.96 (1.27–3.01)	2.46 (1.60–3.79)	< 0.001
IS	161	248	300	395	
Incidence, per 1000 person-year	0.95 (0.81–1.10)	1.58 (1.39–1.78)	1.20 (1.78–2.24)	2.77 (2.51–3.06)	
Model 1	Reference	1.46 (1.20–1.78)	1.71 (1.42–2.08)	2.22 (1.85–2.67)	< 0.001
Model 2	Reference	1.26 (1.03–1.54)	1.26 (1.03–1.53)	1.23 (1.01–1.51)	0.281
Revascularization therapy	74	140	196	356	
Incidence, per 1000 person-year	0.43 (0.35–0.54)	0.89 (0.75–1.05)	1.30 (1.13–1.49)	2.49 (2.24–2.76)	
Model 1	Reference	1.71 (1.29–2.27)	2.24 (1.71–2.92)	3.87 (3.01–4.98)	< 0.001
Model 2	Reference	1.42 (1.07–1.89)	1.58 (1.20–2.07)	2.37 (1.81–3.10)	< 0.001
All-cause mortality	243	321	341	487	
	1.42 (1.25–1.61)	2.03 (1.82–2.26)	2.25 (2.03–2.51)	3.38 (3.09–3.70)	
Model 1	Reference	1.25 (1.06–1.48)	1.28 (1.09–1.52)	1.79 (1.53–2.09)	< 0.001
Model 2	Reference	1.18 (1.00-1.40)	1.16 (0.98–1.38)	1.39 (1.17–1.65)	0.003

Model 1 was adjusted for age and sex

Model 2 was adjusted for age, sex, educational background, smoking status, drinking status, snoring, BMI, SBP, history of hypertension, history of diabetes mellitus, family history of CVD, use of antidiabetic agents, lipid-lowering agents, antihypertensive medications, TC, HDL-C, LDL and hs-CRP and the TyG index

*ASCVD* atherosclerotic cardiovascular disease, *MI* myocardial infarction, *IS* ischemic stroke, *BMI* body mass index, *SBP* systolic blood pressure, *CVD* cardiovascular disease, *TC* total cholesterol, *HDL-C* high density lipoproteincholesterol, *LDL-C* low density lipoproteincholesterol, *hs-CRP* high sensitivity C-reactive protein, *TyG index* triglyceride-glucose index

Cox regression analysis showed that, compared to Q1, the adjusted HR (95% CI) for composite ASCVD events in Q2–Q4 were 1.30 (1.11–1.52), 1.34 (1.15–1.57), and 1.61 (1.38–1.89), respectively (*P* for trend < 0.001). The adjusted HR (95% CI) for all-cause mortality in Q2–Q4 were 1.18 (1.00-1.40), 1.16 (0.98–1.38) and 1.39 (1.17–1.65), respectively (*P* for trend = 0.003). In the analysis of ASCVD event subtypes, a higher TyG index (Q4) was significantly associated with higher risks of MI (HR: 2.46, 95% CI 1.60–3.79) (*P* for trend < 0.001) and revascularization therapy (HR: 2.37, 95% CI 1.81–3.10) (*P* for trend < 0.001). However, a higher TyG index was only significantly associated with a higher risk of IS in model 1 (HR: 2.22, 95% CI 1.85–2.67) (*P* for trend < 0.001) (Table 2).

**Fig. 3:**
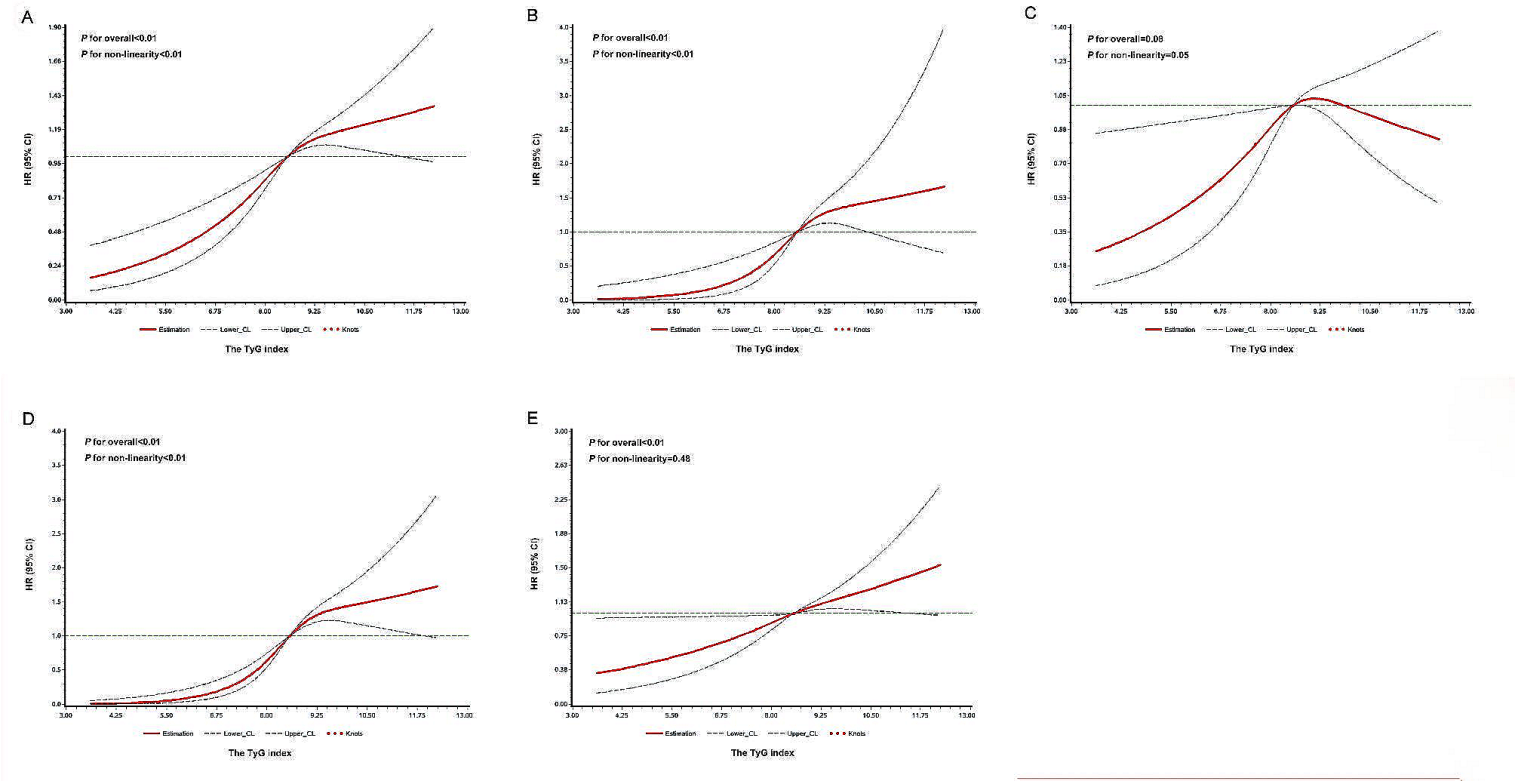
Restricted cubic splines for the association of TyG index with **A** ASCVD events, **B** MI, **C** IS, **D** revascularization therapy, **E** all-cause mortality. **A**–**E** adjusted for age, sex, education, smoking status, drinking status, snoring, BMI, SBP, history of hypertension, history of diabetes mellitus, family history of CVD, use of antidiabetic agents, lipid-lowering agents, antihypertensive medications, TC, HDL-C, LDL-C, hs-CRP and the TyG index. Data were fitted using a Cox regression model of restricted cubic spline with three knots (at the 5th, 50th, and 95th percentiles). Adjusting for potential covariates. The reference point for the TyG index was the median of the reference group. Red lines indicate adjusted hazard ratios, and black lines indicate the 95% confidence interval bands

Restricted cubic spline analysis showed that there was a non-linear relationship between the TyG index and the risk of ASCVD events or MI (*P* for non-linear < 0.01), while there was a linear association between the TyG index and all-cause mortality (*P* for non-linear = 0.476). However, a significant association between TyG index and IS was not confirmed (Fig. 3).

### Sensitivity analysis

The sensitivity analysis between the TyG index and incidence of ASCVD events is shown in Table 3. When the TyG index was considered a continuous variable, the risk of ASCVD events increased by 20% (HR: 1.20, 95% CI 1.12–1.28, *P* < 0.05) and the risk of all-cause mortality increases by 16% (HR: 1.16, 95% CI 1.07–1.26, *P* < 0.05) with every increase of 1 unit in the TyG index. After excluding participants who occurred events at the first 2-year follow-up, the fully adjusted risk of composite ASCVD events, ASCVD subtypes events and all-cause mortality remained consistent with the main model.

**Table 3:** Sensitivity analysis of the association between the TyG index and ASCVD events or all-cause mortality

Variables	Per unit increase	Quartile 1	Quartile 2	Quartile 3	Quartile 4	*P* for trend
ASCVD events	1.20 (1.12–1.28)	Reference	1.25 (1.06–1.49)	1.35 (1.15–1.60)	1.63 (1.06–1.49)	< 0.001
MI	1.37 (1.16–1.62)	Reference	1.60 (0.95–2.67)	2.44 (1.51–3.95)	2.75 (1.69–4.48)	< 0.001
IS	1.05 (0.96–1.16)	Reference	1.20 (0.97–1.49)	1.21 (0.98–1.50)	1.23 (0.99–1.53)	0.062
Revascularization therapy	1.41 (1.27–1.57)	Reference	1.40 (1.03–1.90)	1.62 (1.21–2.17)	2.41 (1.81–3.21)	< 0.001
All-cause mortality	1.16 (1.07–1.26)	Reference	1.17 (0.97–1.41)	1.21 (1.00-1.45)	1.43 (1.19–1.73)	0.001

Model was adjusted for age, sex, educational background, smoking status, drinking status, snoring, BMI, SBP, history of hypertension, history of diabetes mellitus, family history of CVD, use of antidiabetic agents, lipid-lowering agents, antihypertensive agents, TC, HDL-C, LDL-C, hs-CRP and the TyG index

*ASCVD* atherosclerotic cardiovascular disease, *MI* myocardial infarction, *IS* ischemic stroke, *BMI* body mass index, *SBP* systolic blood pressure, *CVD* cardiovascular disease, *TC* total cholesterol, *HDL-C* high density lipoproteincholesterol, *LDL-C* low density lipoproteincholesterol, *hs-CRP* high sensitivity C-reactive protein, *TyG index* triglyceride-glucose index

### Subgroup analysis

To further investigate the association between the TyG index and ASCVD events or all-cause mortality across diverse population, we stratified the population based on sex, BMI, history of hypertension, history of diabetes mellitus, smoking status and drinking status. The results showed that the association between the TyG index and ASCVD events were stronger in non-smokers (HR: 1.87, 95% CI 1.53–2.29) compared to smokers (HR: 1.31, 95% CI 1.02–1.67) (*P* for interaction = 0.042), as well as in non-drinkers (HR: 1.91, 95% CI 1.56–2.33) compared to drinkers (HR: 1.25, 95% CI 0.97–1.60) (*P* for interaction = 0.001). In addition, the association between the TyG index and all-cause mortality were stronger in participants with BMI ≥ 28 (HR: 1.50, 95% CI 0.95–2.35) compared to participants with BMI < 28 (HR: 1.43, 95% CI 1.18–1.73) (*P* for interaction = 0.010), as well as in participants without hypertension (HR: 1.60, 95% CI 1.27–2.01) compared to participants with hypertension (HR: 1.11, 95% CI 0.86–1.45) (*P* for interaction = 0.003) (Table 4).

**Table 4:** Subgroup analysis of the association between the TyG index and ASCVD events or all-cause mortality

ASCVD events				All-cause mortality		
Variables	Adjusted HR (95% CI)	*P*-value	*P* for interaction	Adjusted HR (95% CI)	*P*-value	*P* for interaction
Sex			0.105			0.115
Male	1.44 (1.19–1.73)	0.001		1.37 (1.12–1.67)	0.002	
Female	2.28 (1.70–3.06)	< 0.001		1.44 (1.02–2.02)	0.038	
BMI			0.087			0.010
< 28	1.63 (1.37–1.95)	0.027		1.43 (1.18–1.73)	< 0.001	
≥ 28	1.38 (0.98–1.95)	0.065		1.50 (0.95–2.35)	0.081	
Hypertension			0.116			0.003
Yes	1.76 (1.38–2.24)	< 0.001		1.11 (0.86–1.45)	0.414	
No	1.51 (1.22–1.86)	< 0.001		1.60 (1.27–2.01)	< 0.001	
Diabetes mellitus			0.158			0.819
Yes	3.13 (1.01–9.83)	0.044		1.16 (0.47–2.87)	0.747	
No	1.45 (1.24–1.71)	< 0.001		1.32 (1.10–1.58)	0.002	
Smoking			0.042			0.677
Yes	1.31 (1.02–1.67)	0.033		1.52 (1.16–1.98)	0.002	
No	1.87 (1.53–2.29)	< 0.001		1.30 (1.03–1.62)	0.281	
Drinking			0.001			0.363
Yes	1.25 (0.97–1.60)	0.107		1.44 (1.09–1.88)	0.009	
No	1.91 (1.56–2.33)	< 0.001		1.34 (1.07–1.68)	0.010	

Model was adjusted for age, sex, educational background, smoking status, drinking status, snoring, SBP, history of hypertension, history of diabetes mellitus, family history of CVD, use of antidiabetic agents, lipid-lowering agents, antihypertensive medications, TC, HDL-C, LDL-C, hs-CRP and the TyG index

*ASCVD* atherosclerotic cardiovascular disease, *BMI* body mass index, *SBP* systolic blood pressure, *CVD* cardiovascular disease, *TC* total cholesterol, *HDL-C* high density lipoproteincholesterol, *LDL-C* low density lipoproteincholesterol, *hs-CRP* high sensitivity C-reactive protein, *TyG index* triglyceride-glucose index

### Incremental predictive value of the TyG index

When adjusting TG, FBG, or TyG index into the adjusting models on the base of clinical risk factors, the C-index for predicting ASCVD events were 0.7870 (95% CI 0.7776–0.7963) (*P* < 0.001), 0.7877 (95% CI 0.7783–0.7970) (*P* < 0.001), and 0.7883 (95% CI 0.7790–0.7976) (*P* < 0.001); the category-free NRI were 0.1021 (95% CI 0.0588–0.1454) (*P* < 0.001), 0.0455 (95% CI 0.0009–0.0902) (*P* = 0.0459), and 0.1607 (95% CI 0.1160–0.2054) (*P* < 0.001); the IDI are < 0.0001 (95% CI 0–0.0010) (*P* = 0.3513), 0.0003 (95% CI 0–0.0.0006) (*P* = 0.0198), and 0.0004 (95% CI 0.0002–0.0006) (*P* = 0.0003). Although the C-index for predicting ASCVD events in all models were statistically significant, a statistic significance of the IDI by adjusting TG on the base of clinical risk factors was not confirmed.

When adjusting TG, FBG, or TyG index into the adjusting models on the base of clinical risk factors, the C-index for predicting all-cause mortality are 0.7135 (95% CI 0.7007–0.7263) (*P* < 0.001), 0.7138 (95% CI 0.7011–0.7266) (*P* < 0.001), and 0.7143 (95% CI 0.7015–0.7271) (*P* < 0.001); the category-free NRI are 0.0784 (95% CI 0.0275–0.1293) (*P =* 0.0038), − 0.0309 (95% CI − 0.0837–0.0218) (*P* = 0.2536), 0.1235 (95% CI 0.0705–0.1766) (*P* < 0.001); the IDI are < 0.0001 (95% CI 0–0.0001) (*P* = 0.0890), 0.0010 (95% CI 0.0005–0.0015) (*P* < 0.001), and 0.0003 (95% CI 0.0002–0.0005) (*P* < 0.001). Similarly, although the C-index for predicting all-cause mortality in all models were statistically significant, a statistic significance of the IDI by adjusting TG, the category-free NRI by adjusting FBG were not confirmed (Table 5).

**Table 5:** Incremental predictive value of the TyG index for ASCVD events and all-cause mortality

	C-statistic (95% CI)	*P*-value	Category-free NRI (95% CI)	*P*-value	IDI (95% CI)	*P*-value
ASCVD events						
Clinical risk factors	0.7866 (0.7773–0.7960)		–	–	–	–
Clinical risk factors + TG	0.7870 (0.7776–0.7963)	< 0.001	0.1021 (0.0588–0.1454)	< 0.001	< 0.0001 (0–0.0010)	0.3513
Clinical risk factors + FBG	0.7877 (0.7783–0.7970)	< 0.001	0.0455 (0.0009–0.0902)	0.0459	0.0003 (0–0.0.0006)	0.0198
Clinical risk factors + TyG index	0.7883 (0.7790–0.7976)	< 0.001	0.1607 (0.1160–0.2054)	< 0.001	0.0004 (0.0002–0.0006)	0.0003
All-cause mortality						
Clinical risk factors	0.7132 (0.7004–0.7260)		–	–	–	–
Clinical risk factors + TG	0.7135 (0.7007–0.7263)	< 0.001	0.0784 (0.0275–0.1293)	0.0038	< 0.0001 (0–0.0001)	0.0890
Clinical risk factors + FBG	0.7138 (0.7011–0.7266)	< 0.001	− 0.0309 ( − 0.0837–0.0218)	0.2536	0.0010 (0.0005–0.0015)	< 0.001
Clinical risk factors + TyG index	0.7143 (0.7015–0.7271)	< 0.001	0.1235 (0.0705–0.1766)	< 0.001	0.0003 (0.0002–0.0005)	< 0.001

*ASCVD* atherosclerotic cardiovascular disease, *TG* triglyceride, *FBG* fasting bloodglucose, *TyG index* triglyceride-glucose index, *BMI* body mass index, *SBP* systolic blood pressure, *CVD* cardiovascular disease, *TC* total cholesterol, *HDL-C* high density lipoproteincholesterol, *LDL-C* low density lipoproteincholesterol, *hs-CRP* high sensitivity C-reactive protein

## Discussion

For the first time, we revealed that a higher TyG index is significantly correlate to both early-onset ASCVD events and all-cause mortality in a young and middle-aged population from North China. Indeed, the association of the TyG index with early-onset ASCVD events and all-cause mortality in a young and middle-aged population has not been fully elucidated in previous studies. Xu et al. studied 4,754 African American and white adults aged from 18 to 30 years from the CARDIA study. During a median 25-year follow-up, 158 incident CVD events and 246 cases of all-cause mortality were reported, respectively. The results showed that for each one-unit increase in the TyG index, there was a 96% higher risk of CVD (HR: 1.96, 95% CI 1.44–2.66) and an 85% higher risk of all-cause mortality (HR:1.85, 95% CI 1.45–2.36). Individuals in the high TyG index trajectory group had a significantly increased risk of CVD events (HR: 2.35, 95% CI 1.34–4.12) and all-cause mortality (HR: 3.04, 95% CI 1.83–5.07) compared to those in the low TyG index trajectory group [16]. Cho et al. studied 6,675,424 Korean adults aged from 20 to 39. Over an average follow-up period of 7.4 years, there were 8,506 cases of stroke, 12,312 cases of MI, and 22,667 deaths recorded. The highest TyG index quartile indicated a higher risk of stroke (HR: 1.253, 95% CI 1.167–1.346), MI (HR: 1.258, 95% CI 1.187–1.334), and mortality (HR: 1.151, 95% CI 1.104–1.200) compared to those in the lowest TyG index quartile [17].

However, the results of our current study indicated a weak association between TyG index and IS. Inconsistent with our results, Xu et al. studied 35,999 participants from the Kailuan study. The average age of the participants was 30.8 ± 5.7 years, with 77.1% being male. Over a median follow-up period of 11 years, there were 281 stroke events, including 62 cases of hemorrhagic stroke (HS) and 219 cases of IS. After adjusting for relevant confounders in the Cox proportional hazards model, it was found that subjects with higher TyG index groups had significantly higher risks of early-onset stroke [17]. The reason for our deviating findings probably depends on the following points. First, Xu included the participants aged less than 40 years, which are much younger than those in our study. Second, the study outcome in Xu’s study was stroke in broad terms, which consists both HS and IS, however, we only took IS into consideration in our current study. Third, the relevant confounders adjusted in Cox proportional hazard models were the different.

In addition, our results showed that the association between the TyG index and ASCVD events were stronger in non-smokers than smokers, as well as in non-drinkers compared to drinkers. In addition, the association between the TyG index and all-cause mortality were stronger in participants with BMI ≥ 28 than participants with BMI < 28, as well as in participants without hypertension than participants with hypertension. A special attention should be paid for people with those characteristics. However, because the Kailuan group is a company in the coal industry, considerably more males than females were employed, it is unclear whether this finding is applicable to in a general population.

Furthermore, we demonstrated that although the C-index for predicting ASCVD events or all-cause mortality in all models were statistically significant, a statistic significance of the IDI and the category-free NRI was only confirmed by adjusting TyG index rather than TG or FBG alone. Regarding this, Xu et al. assessed the predictive value of a pooled cohort equations model. The results showed that the C index increased from 0.773 to 0.776 (*P* < 0.001) when taking TyG index into consideration. Moreover, there was a notable improvement in discriminatory power and risk reclassification, with an IDI of 0.010 (95% CI 0.004–0.017) and an NRI of 0.231 (95% CI 0.074–0.389) [16]. In addition, Yao et al. demonstrated that the C-indices of models incorporating TyG for all-cause/non-CV deaths exhibited notably higher predictive values for non-cardiovascular mortality compared to models utilizing TG or FBG alone [18].

Our study has several limitations. First, since the population studied is Chinese, the conclusion applies to the population of Chinese origin. Furthermore, our participants were mainly workers in coal mines in northern China with a higher proportion of males in the population. Therefore, it remains uncertain if the present results are applicable to a general young and middle-aged population. Second, although our results indicated that TyG index improves the prediction of early-onset ASCVD events or all-cause mortality compared to TG and FBG alone, a comparison between TyG index and other IR indices such as homeostatic model assessment for IR was not conducted in our study.

## Conclusions

Our study showed that the TyG index is associated with a high risk of early-onset ASCVD events or all-cause mortality in a young and middle-aged population from North China.

## Data Availability

No datasets were generated or analysed during the current study.

## Abbreviations

ASCVD: Atherosclerotic cardiovascular disease
CVD: Cardiovascular disease
TyG: Triglyceride–glucose
IR: Insulin resistance
